# Differences in PARP Inhibitors for the Treatment of Ovarian Cancer: Mechanisms of Action, Pharmacology, Safety, and Efficacy

**DOI:** 10.3390/ijms22084203

**Published:** 2021-04-19

**Authors:** Giorgio Valabrega, Giulia Scotto, Valentina Tuninetti, Arianna Pani, Francesco Scaglione

**Affiliations:** 1 Department of Oncology, School of Medicine, University of Torino, 10124 Torino, Italy; giulia.scotto@ircc.it (G.S.); valentina.tuninetti@ircc.it (V.T.); 2 Candiolo Cancer Institute, FPO-IRCCS, 10060 Candiolo, Italy; 3 Department of Oncology and Hemato-Oncology, School of Medicine, University of Milan, 20122 Milan, Italy; arianna.pani@unimi.it (A.P.); francesco.scaglione@unimi.it (F.S.)

**Keywords:** ovarian cancer, olaparib, niraparib, rucaparib, safety, efficacy

## Abstract

Poly(ADP-ribose) polymerases (PARP) are proteins responsible for DNA damage detection and signal transduction. PARP inhibitors (PARPi) are able to interact with the binding site for PARP cofactor (NAD+) and trapping PARP on the DNA. In this way, they inhibit single-strand DNA damage repair. These drugs have been approved in recent years for the treatment of ovarian cancer. Although they share some similarities, from the point of view of the chemical structure and pharmacodynamic, pharmacokinetic properties, these drugs also have some substantial differences. These differences may underlie the different safety profiles and activity of PARPi.

## 1. Introduction

Poly(ADP-ribose) polymerase inhibitors (PARPi) are drugs used for cancer treatment that have been approved in recent years. The first approved was olaparib. Later, other molecules with the same mechanism of action arrived. Olaparib, niraparib, and rucaparib are approved for the maintenance treatment of ovarian cancer following completion of first-line platinum-based chemotherapy [1,2,3]. Despite sharing the same mechanism of action, the toxicity profile is different for the three molecules [4,5,6,7,8,9]. Differences in adverse effects of these drugs could be related to dose schedule, half-life, drug interactions, and metabolism. As well as reflecting the different potency of the molecules [10,11], the selectivity toward members of the PARP family and the differences in off-target effects. The purpose of this review is to summarize the pharmacokinetics, pharmacodynamics, efficacy, and safety evidence of olaparib, niraparib, and rucaparib, the three globally approved PARPi in ovarian cancer. In particular, we will focus on the differences between molecules and provide practical suggestions for therapy tailoring.

## 2. Mechanism of Action

Poly(ADP-ribose) polymerases are a family of proteins involved in different cellular processes included DNA repair and apoptosis induction [12]. PARP-1 is the most important member of the family of 18 proteins and is importantly implied in DNA repair [13]. Particularly PARP is involved in the pathways of repair of DNA single-stranded breaks (SSB) and base excision repair (BER) [14,15,16].

PARP activity is triggered by the breakdown of DNA strands. When PARP detects an SSB, it binds to DNA, and after structural changes, it begins the synthesis of poly ADP-ribose (PAR) chains which act as a signal for other DNA repair enzymes [17]. Cleavage of NAD+ substrates with the release of nicotinamide is needed to generate the ADP-ribose monomers.

Furthermore, PARP is able to act on the DSB repair pathway [18] through the modulation of the enzymes MRE11 and NBS1, which are key factors of another important pathway of DNA repair, which is homologous recombination (HR) [19,20,21]. In addition, PARP regulates RAD51 genes through Fbh1 dependent and interacts with and poly-ADP-ribosylates (also known as PARsylates) BRCA1. PARsylation is directed at the BRCA1 DNA binding domain and down-modulates its function. The BRCA1 and interacts with and poly-ADP-ribosylates (also known as PARsylates) BRCA1. PARsylation is directed at the BRCA1 DNA binding domain and down-modulates its function [22,23,24].

PARPi action is based on the concept of synthetic lethality [12]. Synthetic lethality is defined as a condition where the individual loss of two different genes is compatible with life, but when the loss occurs simultaneously for both genes, the cell is unable to survive. In 1997, Hartwell and colleagues first suggested the possibility of using synthetic lethality as a potential strategy for cancer treatment [25]. Since the development of cancer is driven by mutation, often caused by a deficiency in the ability to repair DNA damage [26], it was logical, therefore, to suppose that the pharmacological inhibition of a second pathway involved in DNA repair would have represented a successful strategy.

**Figure 1:**
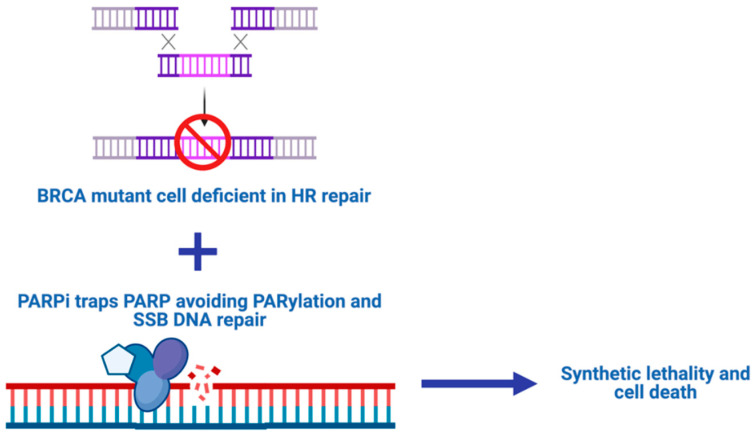
PARP-associated synthetic lethality. PARPi activity is based on the concept of synthetic lethality. BRCA mutant cells are deficient in the HR pathway when a PARPi is associated. In addition, SSB repair is inhibited and apoptosis is induced.

In this perspective, PARPi was born. The synthetic lethality of PARPi is directed against breast-related cancer antigens (BRCA) mutations. In fact, they are able to prevent the repair of DNA single-stranded breaks (SSB) and facilitate the formation of double-stranded breaks (DSB). Since BRCA mutant cells are deficient in the homologous recombination (HR) repair mechanism, the simultaneous inhibition of DNA repair induced by PARPi can lead to cell death through apoptosis (Figure 1). DePARylation is equally important for proper cell function as PARylation. Complete coordination of the two processes is essential for proper DNA damage response (DDR). The function of PARylation is to mediate the recruitment of DDR factors to the proximity of DNA lesions, whereas dePARylation releases these DDR factors from PAR chains, therefore eliciting their engagement at proper DNA lesions for repair. Suppression of dePARylation traps DDR factors onto the PAR chains thus impairs the poly(ADPR) glycohydrolase (PARG)SSB and DSB repair. The most important enzyme involved in dePARylation is that which catalyzes the hydrolysis of ADPR polymer. Targeting of dePARylation may circumvent some of the problems associated with PARPi resistance [27].

Furthermore, PARPi were designed to act together with chemotherapy or radiotherapy responsible for the DNA damage. PARPi would have then prevented the repair of the chemotherapy/radiotherapy-induced DNA damage [28,29,30,31,32].

PARPi have been approved since 2015. Currently, The European Medicines Agency (EMA) has approved rucaparib, olaparib, and niraparib veliparib talazoparib. Interestingly, veliparib was approved for the treatment of NSCLC and SCLC and talazoparib for the treatment of breast cancer.

All PARPi are able to interact with the binding site of the NAD+ located in the catalytic domain of PARP [12,33]. Other than directly inhibiting the repair activity of PARP by competing with NAD+ binding, PARPi are able to trap PARP1 at the level of the SSB and thus prevent the repair [34]. Rucaparib, olaparib, niraparib, and talazoparib especially are able to do this. PARP1 trapping is the basis for PARPi cytotoxicity [34].

A pharmacodynamic difference between clinically used PARPi lies exactly in the different ability to trap PARP1. Talazoparib is the most potent, with the ability to trap PARP1 100 times more efficiently than niraparib. Niraparib is, in turn, more potent than olaparib and rucaparib.

PARPi inhibits PARP1 and other members of the PARP family. However, it is unclear how targeting other members of the PARP family can contribute to the effect exerted by PARPi [14,35,36].

Finally, PARPs functions, especially PARP1, are numerous and involve transcription [37], apoptosis regulation, and immunity modulation other than DNA repair [38,39,40]. Thus, the antitumor action of PARPis could also be associated with these functions.

## 3. Pharmacology

From a pharmacological point of view, PARPi shares some similarities but are different in many characteristics. Regarding pharmacodynamic, all PARPi are able to bind to the NAD+ binding site through a benzamide core pharmacophore. However, they are different in size and flexibility of the molecule. Studies have demonstrated that at micromolar concentrations, clinically used PARPi differ for the capacity of DNA strand break repair, apoptosis induction, and protein phosphorylation, other than the aforementioned different ability to trap PARP [10,41]. In addition, an off-target kinase activity has been described for PARPi, and relevant differences about polypharmacology have been found for the different molecules: niraparib and rucaparib have shown the capacity to inhibit important kinases such as DYRK1s, CDK16, and PIM3 at concentrations achievable with therapeutic doses [42]. Finally, PARPi have different binding affinity for the different PARP family members, as reported in Table 1. These differences are surely implied in the different safety profiles of PARPi.

Moreover, pharmacokinetics differs from one PARPi to another. For example, niraparib is not metabolized by hepatic cytochromes and has less potential for drug-drug interactions [43,44]. Furthermore, the absorption is not influenced by food [42].

Pharmacokinetic characteristics of PARPi are summarized in Table 2.

Olaparib is available in the form of tablets of 150 mg and should be taken twice a day for a total dose of 600 mg. Absorption is rapid, and the individual patient range for Tmax was 0.5–4.2 h for a single dose in a population of Japanese patients with advanced solid tumors. In a multiple dosing regimen, Tmax values were similar to the single-dose administration 1.5–2.1 h, and the individual patient range was 1.5–6.2 h [45]. There is no accumulation after multiple doses, and steady-state is achieved after 3 to 4 days. Plasma concentrations present a biphasic decrease with a mean terminal half-life of 7 to 11 h [1]. After the administration of radiolabeled olaparib, 86% of the total radioactivity was found within 7 days, half in urines and half in feces.

Olaparib is extensively metabolized by CYP3A4/5 [1]. Metabolism of olaparib is attributed to hydroxylation, bis-hydroxylation, hydrolysis, dealkylation, dehydrogenation, and alcohol oxidation. A recent study of in vitro metabolism of olaparib in liver microsomes has identified 12 different metabolites [46].

The association of olaparib with strong or moderate CYP3A inhibitors is not recommended since studies have shown an increase in Cmax of 42% and in AUC of 170% when co-administered. In addition, co-administration with CYP3A inducers is not recommended since the association has shown to decrease mean Cmax by 71% and mean AUC of 87%. Olaparib has also been shown to be a mild CYP3A inducer. It is also possible the clinically relevant induction by olaparib of CYP1A2, 2B6, 3A4, 2C9, 2C19, and P-glycoprotein (P-gp) [1].

When administered with tamoxifen, exposure of olaparib was slightly decreased with Cmax and AUC, respectively, decreased by 20% (90% CI 0.71–0.90) and 27% (0.63–0.84) [47].

Niraparib is commercialized as 100 mg capsules. Posology is 200 mg or 300 mg daily, depending on body weight and platelet count. It has a very rapid absorption (30 min), and Cmax is reached in 3 h. Accumulation following multiple doses is 2 to 3 folds. Plasma exposure increase of niraparib is dose-proportional [3]. Half-life ranged from 48 to 51 h, and the elimination is mainly through the hepatobiliary and renal route [42].

Niraparib is a substrate of carboxylesterases and UDP-glucuronosyltransferases, and the major metabolite M1 is an inactive one [3].

Studies with radiolabeled niraparib have shown a recovery of 47.5% of the drug in urines and 38.8% in feces [44].

Rucaparib is available as 200, 250, or 300 film-coated tablets, and the daily dose is 1200 mg. Pharmacokinetic of rucaparib has been evaluated in patients with advanced solid tumors in the phase I/II study [48]. Median Tmax ranges between 1.5–6.0 h. Steady-state was reached after 1 week with four-fold accumulation. Plasma exposures of rucaparib were dose-proportional for every evaluated dose. After 600 mg daily dosing Cmax was 16,900 ng/mL with rapid absorption (Tmax ~1.9 h) [2].

According to in vitro studies, rucaparib is metabolized by CYP2D6, CYP1A2, and CYP3A4. Oxidation, N-demethylation, N-methylation, glucuronidation, and N-formylation were the major metabolic pathways. The major metabolite of rucaparib is the M324 result of the oxidative metabolism of rucaparib [49]. Other six minor metabolites have been identified. Studies with radiolabeled rucaparib showed that rucaparib and M324 were the major rucaparib-related components found in urines, while rucaparib was the predominant component found in feces [50].

Rucaparib is a moderate inhibitor of CYP1A2 and a mild inhibitor of CYP2C9, CYP2C19, and CYP3A. It is also a weak inhibitor of CYP2C8, CYP2D6, and UGT1A1 [2,51].

Rucaparib could be a substrate of P-gp; thus, it is suggested caution when co-administering strong P-gp inhibitors [2].

## 4. Efficacy and Safety of Olaparib

The first PARPi approved in the clinic was olaparib, based on the results of the SOLO2 study [52]. In this randomized placebo-controlled phase 3 clinical trial 295 BRCA-mutated patients affected by a platinum-sensitive relapse of ovarian/fallopian tube/primitive peritoneal cancer, already treated with at least two platinum-based lines, were assigned to maintaining with olaparib (formulation of tablets at a dose of 300 mg twice a day) versus placebo. The advantage offered by PARPi in terms of progression-free survival (PFS) was 13 months higher than the placebo arm (19.1 months versus 5.5 months; *p* < 0.0001).

At the last congress of the American Society of Clinical Oncology (ASCO) was presented the data of overall survival (OS) of the study SOLO2, the first data of survival concerning a PARPi: the addition of olaparib revealed a statistically significant survival advantage of 12.9 months compared to placebo (51.7 months against 38.8; HR 0.74; *p* = 0.054), an unprecedented result [53,54].

The scenario of first-line medical therapy of ovarian cancer has been radically modified by the phase 3 SOLO1 study, a randomized and placebo-controlled trial in which olaparib was evaluated as maintenance therapy in 391 patients with ovarian cancer newly diagnosed in an advanced stage with somatic or germline BRCA mutations who had responded platinum-based chemotherapy. In BRCA-mutated patients, there was a rate of PFS at 3 years of 60% and a reduction in the risk of progression or death of 70% (HR 0.30) [55].

PAOLA-1 is a randomized and controlled phase 3 trial, in which, after response to platinum-based first-line chemotherapy, patients with ovarian cancer, regardless of the presence or absence of BRCA mutations, were divided into two arms: olaparib and bevacizumab versus placebo and bevacizumab. The combination of the two drugs significantly prolonged PFS in the entire population, with a 41% reduction in the risk of progression or death in the arm treated with olaparib (HR 0.59; P 0.001) [56]. However, the study schema lacked a control arm with olaparib alone, so the PAOLA-1 trial could not clarify whether olaparib and bevacizumab have a synergistic effect, making it difficult to understand which patients might benefit most from the combination. EMA, on the basis of PAOLA-1 results, approved olaparib in combination with bevacizumab for the maintenance treatment of adult patients with FIGO stages III and IV high-grade epithelial ovarian cancer who are in response following completion of first-line platinum-based chemotherapy and whose cancer is associated with homologous recombination deficiency (HRD) positive status defined by either a BRCA1/2 mutation and/or genomic instability assessed with the Myriad myChoice^®^ test which evaluates loss of heterozygosity (LOH), telomeric allelic imbalance (TAI), and large-scale state transitions (LST) using DNA isolated from formalin-fixed paraffin-embedded (FFPE) tumor tissue specimens [53,56,57,58,59].

**Figure 2:**
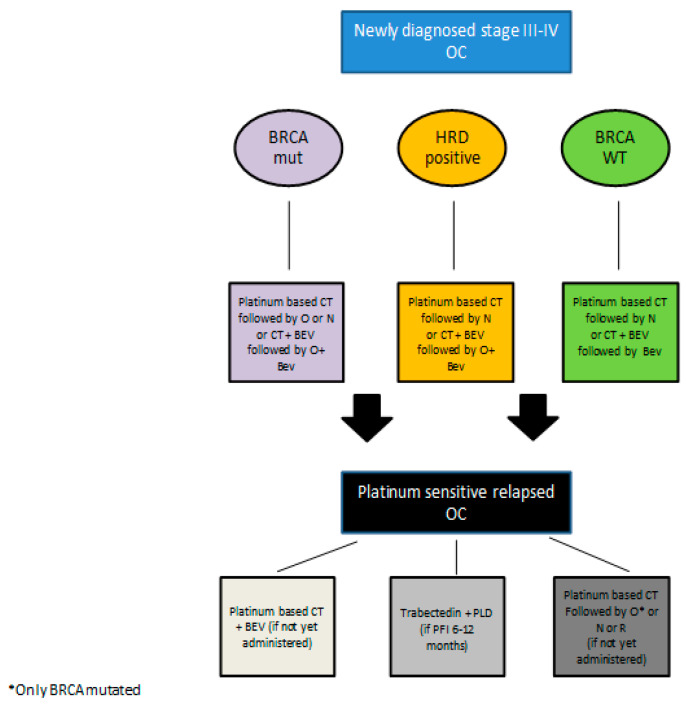
Proposed therapeutic algorithm for the treatment of advanced and relapsed epithelial ovarian cancer according to EMA indication. Legend: CT = chemotherapy; O = olaparib; N = niraparib; BEV = bevacizumab; PFI = platinum free interval; R = rucaparib.

See Figure 2 for a proposed therapeutic algorithm including olaparib according to EMA indications.

Overall in phase III randomized trials, adherence to olaparib treatment is very good and consistent with that observed in study 19 [60,61,62,63]. The most frequently (≥10%) observed adverse reactions across clinical trials in patients receiving olaparib monotherapy were nausea, fatigue, vomiting, diarrhea, dysgeusia, headache, abdominal pain, decreased appetite, constipation, cough, arthralgia, dizziness, dyspepsia, pyrexia, dyspnea, anemia, neutropenia, thrombocytopenia, leukopenia [55].

We report below data from the SOLO-2 trial.

The grade ≥3 adverse reactions occurring in >2% of patients were anemia (19%), neutropenia (5%), fatigue/asthenia (4%), nausea (3%), vomiting (3%), abdominal pain (3%), leukopenia (2%) and thrombocytopenia (3%) [52,55].

Regarding gastrointestinal toxicities, in the SOLO2 study, 73.3% of patients experienced grade 1–2 nausea (versus 33.3% in the placebo group), but only 2.6% of patients complained of grade 3 nausea. Only 2.6% of patients complained of grade 3 vomiting, and similar results are reported in the SOLO1 trial [52,55,64].

Although dyspnea may be a sign of anemia, it is important to consider the rare possibility of pneumonitis (<1% incidence in clinical studies) in patients with dyspnea, cough, or fever, or associated abnormal chest radiologic findings. If pneumonitis is confirmed, olaparib treatment should be discontinued, and the patient treated appropriately.

Peculiar toxicity is the onset of myelodysplastic syndrome/acute myeloid leukemia (MDS/AML), although the incidence is rare (1% in the SOLO1 study) [55]. Long-term follow-up of the SOLO2 trial (>5 years in each group) showed 16 cases of MDS/AML in the olaparib group and four cases in the placebo group (*p-*value not reported) [53]. This increase in incidence is probably due to the fact that patients undergoing maintenance with the PARPi at relapse are treated continuously, likely until disease progression; therefore, given the survival advantage offered by these drugs, treatment can continue for several years, and this may expose the patients to an increased risk of MDS/AML.

## 5. Efficacy and Safety of Niraparib

In the pivotal phase 3 NOVA trial, 553 women with platinum-sensitive ovarian cancer relapse, categorized according to the presence or absence of a germline BRCA mutation (gBRCA cohort and non-gBRCA cohort), were randomly assigned in a 2:1 ratio to receive niraparib (300 mg) or placebo once daily. Myriad myChoice^®^ test was used to test HRD. In the cohort with gBRCA, there was an advantage of PFS associated with niraparib of almost 16 months (21 months against 5.5 months; HR 0.27; *p* < 0.0001), as compared with 12.9 months versus 3.8 months in the non-gBRCA cohort for patients who had tumors with HRD (HR 0.38; *p* < 0.0001) while in the cohort without gBRCA the advantage was about 6 months (9.3 months vs. 3.9, HR 0.45; *p* < 0.001) [65].

PRIMA is a randomized and placebo-controlled phase 3 trial aimed at assessing the efficacy of niraparib as maintenance therapy in patients with newly diagnosed advanced ovarian cancer after the response to platinum-based first-line therapy. In the population with HRD (51% of patients in the study), maintenance therapy with niraparib was associated with a 57% reduction in the risk of progression or death (HR 0.43, CI 95% 0.31 to 0.59; *p* < 0.001), while in the intention-to-treat (ITT) population the risk reduction was 38% (HR 0.62). In the subgroup analysis, HRD-positive patients, but not BRCA-mutated, benefit from maintenance with PARP inhibitor almost as much as BRCA-mutated patients (HR for PFS 0.50 and 0.40, respectively); in addition, treatment with niraparib has been shown to provide a clinically significant benefit also in the HR-proficient subgroup, although lower than that observed in the two subgroups mentioned above, with a 32% reduction in the risk of progression or death (HR 0.68) [66].

See Figure 2 for a proposed therapeutic algorithm including niraparib according to EMA indications.

Incidence and severity of side effects in PRIMA and NOVA trials were similar, and globally, adherence to treatment was very good [67,68,69].

Above, we report more in detail the ones from the NOVA trial.

The most frequently (≥10%) observed any grade adverse reactions in patients receiving niraparib monotherapy were nausea, thrombocytopenia, fatigue, anemia, constipation, vomiting, neutropenia, headache, decreased appetite, insomnia, abdominal pain, dyspnea, hypertension, diarrhea, dizziness, cough, back pain, arthralgia, dyspepsia.

The most common grade 3–4 adverse reactions related to treatment with niraparib (>1% of patients) were thrombocytopenia (33.8%) and anemia (25.3%), neutropenia (19.3%), Hypertension (8.2), fatigue (8.2%), nausea (3%), vomiting 1.9%, Abdominal pain (1.1%), dyspnea (1.1%).

Thrombocytopenia is a peculiar toxicity in treatment with niraparib. The posthoc analysis of the NOVA trial published in Annals of Oncology by Berek and colleagues notes that the most common dose used by patients was 200 mg/day (not 300 mg/day, standard start-up dose) and in particular patients with a weight < 77 kg or platelets count <150,000/uL may benefit from a starting dose of 200 mg/day without differences in efficacy compared to patients treated with a full dose [70].

On the basis of these results, the PRIMA study was amended to individualize starting dose based on body weight (> or <77 kg) and platelet count (> or <150,000/µL, or both) with patients starting at 300 mg if >77 kg and platelets >150,000. PRIMA results of adverse reactions (RADAR) analysis were presented at ESMO 2018 [70]. A total of 471 patients received a fixed starting dose of niraparib/placebo (300 mg), and 159 patients were treated with an individualized dose of niraparib/placebo according to weight and platelet count (200 mg for bodyweight <77 kg and platelet count <150,000 μL). AEs grade ≥3 were lower in the individualized dosing group (pooled niraparib/placebo) as compared with the group that received a fixed starting dose of 300 mg of niraparib/placebo [71].

Moreover, recently published data from the NORA trial as real-world evidence [68] confirmed that dose individualization is associated with improved hematologic toxicity [6].

Interestingly, in the PRIMA study, one patient in the niraparib group received the diagnosis of myelodysplastic syndrome in the context of bowel perforation, sepsis, and progressive disease [66].

In the NOVA trial, the incidence of MDS/AML was similar between the niraparib group (1.4%) and the placebo group (1.1%) [9,65].

In the PRIMA trial, grade 3–4 hypertension, a peculiar adverse reaction of niraparib, occurred in 6% of niraparib-treated patients (versus 1% of placebo-treated patients) with a median time from the first dose to the first onset of 50 days. Hypertension of grade 3–4 occurred in 8.2% of niraparib-treated patients in the NOVA trial. There are no specific guidelines for niraparib-induced hypertension at this time, but the management should follow standard guidelines, maintaining value below 120/80 mmHg [72].

Another unexpected adverse event is insomnia, very common (incidence ≥ 1/10) in niraparib-treated patients (grade 1–4: 24.3% of niraparib group in NOVA trial; 26.4% in PRIMA trial) [65,66,73].

## 6. Efficacy and Safety of Rucaparib

The latest approved PARPi in the maintenance setting of relapsed ovarian cancer after partial or complete response to platinum-based chemotherapy is rucaparib, which has been tested at a dose of 600 mg twice a day in the ARIEL3 study. This randomized phase 3 trial involved 564 patients, both BRCA-mutated and BRCA wild-type (WT), assigned in 2:1 ratio to treatment with rucaparib or placebo. The study population was divided into three different cohorts: BRCA-mutated, HRD-positive (including BRCA-mutated and those with high loss of heterozygosity, LOH, an indicator of a deficit in homologous recombination), and total ITT (HRD-positive cohort plus those with indeterminate or low LOH values, and therefore considered HRD-negative). The test used to assess LOH was Foundation Medicine’s T5 NGS assay. Maintenance with rucaparib resulted in a statistically significant increase in PFS in all three cohorts, although the highest efficacy was recorded in the BRCA-mutated population (16.6 months versus 5.4 months; HR 0.23; *p* < 0.0001) [33,74,75].

See Figure 2 for a proposed therapeutic algorithm including rucaparib according to EMA indications.

The most frequently (≥10%) observed any grade adverse reactions in patients receiving rucaparib monotherapy were nausea, fatigue, dysgeusia, anemia, constipation, alanine aminotransferase (ALT) or aspartate aminotransferase (AST), diarrhea, abdominal pain, thrombocytopenia, decreased appetite neutropenia, headache, photosensitivity reactions, cough, dizziness, arthralgia, increase in blood creatinine concentration, dyspepsia [76].

The most common grade 3–4 adverse reactions related to treatment with rucaparib (>1% of patients) were anemia (19%), AST or ALT increase (10%), neutropenia (7%), fatigue (7%), thrombocytopenia (5%), nausea (4%), vomiting (4%), constipation (2%), and abdominal pain (2%).

In general, for hematological toxicities, the time of onset was generally delayed (after about 2 months of treatment). In the ARIEL3 study, the incidence of MDS/AML being treated in patients who received rucaparib was 0.8% [74,77].

Interestingly, the increased ALT and AST occurred within the first weeks of treatment with rucaparib, which was reversible and rarely associated with increased bilirubin.

Increases in serum creatinine, mild to moderate (CTCAE grade 1 or 2), were observed in 15% of patients within the first few weeks of treatment with rucaparib. Only one patient (<1%) reported a CTCAE grade 3 reaction. This adverse reaction could be due to the inhibition that rucaparib exerts on renal transporters MATE 1 and MATE2-K. These increases in serum creatinine were clinically asymptomatic.

Photosensitivity has been observed in patients treated with rucaparib. During rucaparib treatment, it is recommended to avoid direct sunlight and to use sunscreen with a sun protection factor (SPF) of 50 or greater.

## 7. Efficacy and Safety of Veliparib

VELIA trial was a phase III, placebo-controlled study, which assessed the efficacy of veliparib added to first-line induction chemotherapy with carboplatin and paclitaxel and continued as maintenance monotherapy in patients with previously untreated stage III or IV high-grade serous OC.

In the overall population, a regimen of carboplatin, paclitaxel, and veliparib followed by veliparib maintenance therapy led to significantly longer PFS than carboplatin plus paclitaxel induction therapy alone. Indeed, in the *BRCA*-mutation cohort, the median PFS was 34.7 months in the veliparib-throughout group and 22.0 months in the control group (HR 0.44; 95%, *p* < 0.001); in the HRD cohort, it was 31.9 months and 20.5 months, respectively (HR 0.57; *p* < 0.001); and in the intention-to-treat population, it was 23.5 months and 17.3 months (HR 0.68; *p* < 0.001).

Veliparib led to a higher incidence of anemia (65%) and thrombocytopenia (60%) when combined with chemotherapy, as well as of nausea (72%) and fatigue overall (62%) [78].

See Table 3 for the most relevant differences in toxicity between the three PARPi.

## 8. Discussion

### 8.1. Differences in Clinical Trial Designs

Although no direct comparisons have been made in the literature between different PARPi, no differences in efficacy seem to have been highlighted considering the endpoints of the studies performed [79,80].

The features of populations included in the various frontline maintenance therapy protocols, however, have differences that should be taken into account while choosing the most appropriate PARPi for each patient. In particular, the patients included in the SOLO1 trial (median follow-up 5 years) [81] were all BRCA-mutated, 80–85% of patients presented FIGO stage III at diagnosis, and 82% achieved a complete response to first-line chemotherapy. This can be considered a good prognosis population [55].

In the PRIMA trial (median follow-up 24 months), however, 35% of patients had FIGO stage IV ovarian cancer at onset (a higher percentage than in the SOLO-1 study), 99.6% of stage III patients had residual disease after primary debulking surgery (PDS), and in 67% of cases, neoadjuvant chemotherapy (NACT) was conducted. In addition, about 30% of the patients achieved a partial response to first-line chemotherapy (versus only 18% in the SOLO1 trial). As for the status of BRCA and HR, 51% of patients were HR-deficient (HRD-positive patients), 30% were BRCA-mutated, while 20% were HR-deficient, but BRCA-WT. HR-proficient patients were at least 30%, although the exact percentage is not known with certainty, because for about 10–15% of the enrolled patients, the material available was not sufficient to complete the HRD test [66]. This population certainly has a worse prognosis than that included in the SOLO1 trial.

In the PAOLA1 study (median follow-up 22.9 months), the situation was intermediate between the previous two studies: in fact, about half of the patients had undergone upfront surgery, with a fair percentage, about 60%, of the absence of macroscopic disease after surgery, just over 40% had undergone interval debulking surgery (IDS), with a percentage of no residual macroscopic disease around 70% and the rest of the patients had only NACT. Furthermore, 48% of patients were HRD-positive and, in this subgroup, 29% were HRD-positive and BRCA-mutated, while 19% were HRD-positive, but with BRCA wild-type; 34% were HRD-positive, and for 18%, the HRD status was unknown [56].

The patient’s HR status is an important element in the choice of PARPi in the first-line maintenance therapy. In fact, olaparib can be prescribed only for BRCA-mutated patients, while niraparib also in BRCA wild-type patients, although it has shown greater efficacy in BRCA-mutated and HRD patients. The combination olaparib/bevacizumab, not yet approved by all European countries, can be prescribed only for HRD patients.

With regard to the trials conducted in the disease relapse setting, there are no substantial differences in the study populations, except for the genetic status: in the SOLO2 trial were included only BRCA-mutated patients. There are similar percentages of platinum-sensible and partial platinum-sensible relapsed ovarian cancers (40% vs. 60% in the three studies), previous bevacizumab about 20–25%, and at least two previous lines of chemotherapy as inclusion criteria.

Only a slight imbalance was found for percentages of responses at the last platinum-based line: SOLO2 and NOVA trial showed 50% of partial responses and 50% of complete responses, while in the ARIEL3 trial were registered 66% of partial responses and 34% of complete responses [52,65,74].

In the relapsed setting, only olaparib had a long enough follow-up to produce the first survival data for a PARPi: in fact, at 5 years, 42.1% of olaparib arm patients versus 33.2% of placebo arm patients were alive, with an improvement of 12.9 months in median OS versus placebo [53]. This element must be taken into consideration when choosing the PARPi in BRCA-mutated patients.

### 8.2. Which One to Choose Based on Toxicity?

Hematological toxicities are common adverse events of PARPi therapy.

Anemia is the most frequent: in the three clinical trials conducted in the recurrence setting, 25% of patients treated with niraparib and 19% of patients treated both with olaparib or rucaparib showed grade 3 and 4 anemia [52,65,74]. In the first-line setting, 31% of patients treated with niraparib and 22% of patients treated with olaparib experienced grade 3 or 4 anemia. It was demonstrated that loss of PARP2 in mice can shorten the lifetime of erythrocytes and impair the differentiation of erythroid progenitors [82].

Thrombocytopenia is more frequent with niraparib (34% grade 3–4 in NOVA trial) than with other PARPi in the relapse setting (5% with rucaparib and 1% with olaparib) [52,65,74]. In the first-line setting grade 3–4, thrombocytopenia rates reported are similar: 29% in PRIMA trial and 1% in SOLO1 trial [55,66]. This side effect seems to be associated with reversible inhibition of megakaryocyte proliferation and maturation [83].

Neutropenia is the third more frequent adverse hematological event, reported in 20% (grade 3–4) of patients treated with niraparib, compared with 7% with rucaparib and 5% with olaparib [52,65,74]. In the PRIMA trial, 13% of patients experienced grade 3–4 neutropenia, while in the SOLO1 trial, 8% of patients treated with olaparib showed grade 3–4 neutropenia [55,66].

In the systematic review and network meta-analysis of Stemmer and colleagues, which included six randomized controlled trials and compared the approved PARPi, niraparib seems to be the most toxic, showing a significant statistical difference in the risk for grade 3–4 thrombocytopenia and any grade neutropenia [79]. Another important feature is the percentage of patients who needed a dose reduction due to adverse events correlated with PARPi therapy: this rate ranging from 25.1% to 28% for olaparib, 54.6% for rucaparib, and 66.5% to 70.9% for niraparib [79].

On the contrary, Mirza et al., comparing the main first-line trials conducted with PARPi, did not show significant differences in toxicity between drugs [80]. In the PRIMA study, the use of the individualized starting dose of 200 mg of niraparib for patients with body weight < 77 kg and/or platelet counts <150,000/µL, based on results of posthoc analysis by Berek et al. [70], may have improved safety profile in the first-line setting.

The differences between the safety profiles of PARPi may be associated with their structural differences and distinct pharmacokinetic properties: niraparib is a selective inhibitor of PARP1 and PARP2, while olaparib and rucaparib are more potent inhibitors of PARP1 but are less selective [84].

Furthermore, Antolin et al. reported the first complete characterization of the off-target kinase of PARPi approved by FDA. They demonstrated that niraparib and rucaparib inhibit DYRK1s, CDK16, and PIM3 at submicromolar concentrations. These kinases are the most mightily inhibited off-targets of PARPi identified and should be more investigated in order to explain some differences between PARPi safety profiles and efficacy in the clinic [42].

Hematological adverse events that correlate to PARPi are frequent but usually transient, occurring during the first months of therapy, and often resolved with dose reduction. However, some rare and serious cases of acute myeloid leukemia and myelodysplastic syndrome have emerged. The recent systematic review and safety meta-analysis of Morice et al. that included 28 randomized controlled trials (RCTs) comparing PARPi therapy in different tumors (more frequently ovarian cancers) with control treatments showed that PARPi significantly increased the risk of MDS/AML compared with placebo (Peto OR 2.63, *p* = 0.026).

They also reported features of PARPi-related MDS/AML cases derived from WHO’s pharmacovigilance database (VigiBase): for the 99 cases of MDS and 79 cases of AML collected, the median latency period since first exposure to a PARP inhibitor was 17.8 months, and 45% of cases resulted in death. Subgroup analysis did not show significant differences among PARPi used, patients with BRCA mutations or BRCA wild-type, PARPi treatment setting, and PARPi treatment duration [85]; however, for olaparib, there is a lack of definitive data for its use in wild-type BRCAs.

Certainly, the limited follow-up of many RCTs could underestimate the real incidence of PARPi-related MDS/AML cases [85,86]. If we consider the final analysis of SOLO2 [53], which showed for the first time a statistically significant advantage in OS with PARPi maintenance therapy, after five years of follow-up, also reported 16 cases of MDS/AML versus only four in the placebo group.

Bolton et al. demonstrated that patients exposed to PARPi therapy were more likely to have clonal hematopoiesis, in particular in the DNA damage response pathway (DDR), compared to those exposed to conventional chemotherapies or untreated patients. This DDR-mutated clonal hematopoiesis could lead to an increased risk of MDS/AML [86].

## 9. Conclusions

Although it is currently clear that PARPi are the maintenance therapy of choice in ovarian cancer, no direct comparison of efficacy has been made between the three main PARPi approved in the clinic. Moreover, even if these are drugs with a manageable toxicity profile, it is important not to underestimate the hematological toxicity and the risk of developing acute myeloid leukemia and myelodysplastic syndrome.

According to the comorbidities and the baseline laboratory exams of each patient, we have some elements better personalize treatments: in particular, for the BRCA wild-type patients, niraparib should be treated with a personalized dose if a patient shows a reduced platelet count (<150,000/µL), bodyweight < 77 kg or severe hypertension. In the presence of elevated AST and or ALT or renal insufficiency at baseline, rucaparib may not be the preferential choice. In the presence of swallowing issues, it should also be considered that the number of niraparib tablets is lower than that of olaparib or rucaparib tablets (full dosage). In conclusion, for equal effectiveness and in the absence of direct comparison studies, the clinician has the possibility to adapt the maintenance therapy on the basis of the specific clinical, laboratory, and genetic features of each patient. We are waiting for follow-up updates of ongoing studies to have more safety information and more solid overall survival results.

## Figures and Tables

**Table 1:** PARPi IC50 values for PARP family members. IC50 values have been obtained from the ChEMBL database.

	Olaparib	Niraparib	Rucaparib
**PARP1**	1–19 nM	2–35 nM	0.8–3.2 nM
**PARP2**	1–251 nM	2–15.3 nM	28.2 nM
**PARP3**	46–230 nM	296–1.3 nM	512 nM
**PARP4**	410 nM	330–446 nM	839 nM

**Table 2:** Pharmacokinetic characteristics of PARP inhibitors.

	Olaparib	Niraparib	Rucaparib
**Posology**	300 bid	300 mg	600 bid
**Bioavailability**	NA	73%	30–45%
**AUC 0-24**	42,000 h ng/mL	NA	1690 h ng/mL
**Cmax**	58,000 ng/mL	3 h	1940 ng/mL
**Tmax**	1–3 h	NA	1.9 h
**Plamatic Clearence**	8.6 L/h	16.5 L/h	13.9–18.4 L/h
**Volume of Distribution**	167 L	1311 L	113–262 L
**Half-life**	11.9 h	48-51 h	25.9 h
**Co-Administration with Food**	Food assumption delays Tmax of about 2 h	No influence	After a highly lipidic meal, Cmax is increased by 20% and AUC of 38%, while Tmax is delayed by 2.5 h
**Plasmatic Protein Binding**	Dose-dependent: bound fraction decreases from 91% at 1 microg/mL concentration to 82% to qo microg/mL and to 70% at 40 microg/mL	83%	70.2%
**Metabolism**	CYP3A4/5 are enzymes primarily responsible for metabolism	Carboxilestherasis are the enzymes primarily responsible for metabolism	CYP2D6 and CYP1A2 e CYP3A4 are the enzymes primarily involved in metabolism
**Substrate of**	P-gp (clinically non-significant)	P-gp, BRCP, MATE1/2 (clinically non-significant)	P-gp and BCRP
**Cytochromes and Transporters Inhibition**	Induction of CYP1A2, 2B6 e 3A4	Inhibition of MATE1/2 e and mild inhibition of OCT1	Moderate inhibition of CYP1A2
**Cytochromes and Transporters Inhibition**	Moderate inhibition of CYP3A, P-gp, BCRP, OATP1B1, OCT1, OCT2, OAT3, MATE1, MATE2K	None	Mild inhibition of CYP2C9, CYP2C19, CYP3A E P-gp
**Renal Impairment**	Severe renal impairment (ClCr < 30 mL/min): not recommended	Severe renal impairment (ClCr < 30 mL/min): not recommended	Severe renal impairment (ClCr < 30 mL/min): not recommended
	Moderate renal impairment (CrCl 31–50 mL/min): dose reduction to 300 mg × 2	Moderate renal impairment (CrCl 31–50 mL/min): no dose adjustment	Moderate renal impairment (CrCl 31–50 mL/min): no dose adjustment
	Mild renal impairment (ClCr 51–80 mL/min): no dose adjustment	Mild renal impairment (ClCr 51–80 mL/min): no dose adjustment	Mild renal impairment (ClCr 51–80 mL/min): no dose adjustment
**Hepatic Impairment**	Mild or moderate hepatic impairment (child pug A or B): no dose adjustment	Mild or moderate hepatic impairment (child pug A or B): no dose adjustment	Mild or moderate hepatic impairment (child pug A or B): no dose adjustment
	Severe hepatic impairment (child pug C): not recommended	Severe hepatic impairment (child pug C): not recommended	Severe hepatic impairment (child pug C): not recommended

**Table 3:** Relevant differential toxicities (G3-4) across olaparib, niraparib, and rucaparib.

	OLAPARIB	NIRAPARIB	RUCAPARIB
**Neutropenia**		●	
**Thrombocytopenia**		●	
**Raised ALT/AST**			●
**Hypertension**		●	
**Tablet Intake**	●		●
**Anemia**	●	●	●
**Fatigue**		●	●
